# Reduced thalamic glutathione in migraine: a MEGA-PRESS spectroscopy study

**DOI:** 10.1186/s10194-026-02379-y

**Published:** 2026-05-04

**Authors:** Hongying Huang, Jiayin Miao, Yinji Piao, Zhaodong Lin, Yuqin Lin, Rui Wang, Zhongruowen Ren, Guangsong Wang, Dafa Shi, Gen Yan, Yongmin Chang, Renhua Wu

**Affiliations:** 1 Department of Radiology, The Second Affiliated Hospital of Xiamen Medical College, Xiamen, China; 2 Department of Radiology, The Second Affiliated Hospital of Shantou University Medical College, Shantou, China; 3 Department of Neurolgy, The Second Affiliated Hospital of Xiamen Medical College, Xiamen, China; 4 Department of Radiology, Sun Yat-Sen Memorial Hospital, Sun Yat-Sen University, Guangzhou, China; 5 Department of Pathology, The Second Affiliated Hospital of Xiamen Medical College, Xiamen, China; 6 Department of Radiology, Xiang’an Hospital of Xiamen University, School of Medicine, Xiamen University, Xiamen, China; 7 Department of Radiology, Kyungpook National University Hospital, Daegu, Republic of Korea; 8 Department of Molecular Medicine, School of Medicine, Kyungpook National University, Daegu, Republic of Korea

**Keywords:** Migraine, Tension-type headache, Glutathione, MEGA-PRESS spectroscopy, Thalamus

## Abstract

**Background:**

Migraine and tension-type headache (TTH) are highly prevalent primary headache disorders lacking objective diagnostic biomarkers. Oxidative stress has been implicated in migraine pathophysiology, with glutathione (GSH) serving as the brain’s principal antioxidant. This study investigated whether thalamic GSH levels, measured using optimized MEGA-PRESS spectroscopy, could serve as a candidate biomarker for differentiating migraine from TTH.

**Methods:**

This cross-sectional study enrolled 76 participants: 20 healthy controls (HC), 19 TTH patients, and 37 migraine patients (27 without aura, 10 with aura). Bilateral thalamic GSH was measured using MEGA-PRESS at 3T. Group comparisons employed one-way ANOVA with Tukey HSD post-hoc tests. Sex-stratified analyses were performed. ROC analysis evaluated diagnostic performance.

**Results:**

Migraine patients showed significantly reduced thalamic GSH compared to both HC and TTH (left thalamus: F = 30.78, *p* < 0.001, η² = 0.457). Critically, no difference was found between HC and TTH groups. Sex-stratified analyses showed that GSH reduction was significant in both males and females. Left thalamic GSH showed good diagnostic accuracy for distinguishing migraine from both HC (AUC = 0.897) and TTH (AUC = 0.879).

**Conclusions:**

Thalamic GSH reduction appears to differentiate migraine from TTH in this sample, supporting a distinct role of oxidative stress in migraine pathophysiology. GSH may serve as a candidate biomarker for differentiating migraine from TTH.

**Clinical trial number:**

Not applicable.

## Background

Migraine is one of the most prevalent neurological disorders, affecting approximately 12% of the global population and ranking as the second leading cause of years lived with disability worldwide [1, 2]. Despite its significant burden, migraine diagnosis remains primarily clinical, relying on patient-reported symptoms according to International Classification of Headache Disorders criteria [3]. The differentiation between migraine and tension-type headache (TTH), another highly prevalent primary headache disorder, can be challenging in clinical practice due to overlapping features [4]. 

The thalamus has emerged as a key structure in migraine pathophysiology, serving as a critical relay station for sensory information including nociceptive signals [5, 6]. Early clinical electrophysiological and imaging work reported that, during migraine attacks with cutaneous allodynia, thalamic neurons exhibit enhanced activation, helping to establish the thalamus as a central node in migraine pathophysiology [7]. Neuroimaging studies have revealed structural and functional alterations in the thalamus of migraine patients, including morphological abnormalities of thalamic subnuclei and altered connectivity patterns [8, 9]. Recent magnetic resonance spectroscopy (MRS) studies have reported neurochemical changes in the thalamus, including altered glutamate/glutamine (GLX) and γ-aminobutyric acid (GABA) levels, suggesting that metabolic disturbances in this region may contribute to migraine pathophysiology [10, 11]. 

Oxidative stress has been increasingly recognized as a potential mechanism in migraine pathogenesis [12, 13]. A recent comprehensive review by Jiménez-Jiménez et al. summarized evidence supporting the role of oxidative stress in migraine, including altered levels of oxidative damage markers and antioxidant enzymes [14]. Mitochondrial dysfunction and increased production of reactive oxygen species (ROS) have been observed in migraine patients, and antioxidant therapies have shown some efficacy in migraine prevention [15, 16]. Glutathione (GSH), a tripeptide consisting of glutamate, cysteine, and glycine, serves as the brain’s primary endogenous antioxidant and plays crucial roles in neutralizing ROS and maintaining cellular redox balance [17, 18]. 

Proton magnetic resonance spectroscopy (^1^H-MRS) enables non-invasive measurement of brain metabolites in vivo and has emerged as a valuable tool for investigating neurochemical alterations in migraine [19, 20]. The MEGA-PRESS technique has been optimized for GSH measurement by targeting the cysteine moiety at 2.95 ppm [21, 22]. Zhang et al. recently reported that occipital GSH levels were reduced during migraine attacks compared to the interictal period, suggesting dynamic changes in oxidative stress across the migraine cycle [23]. However, no study has specifically compared thalamic GSH between migraine and TTH patients, leaving the specificity of GSH alterations to migraine unclear.

There are a priori reasons to consider that oxidative stress may play a different role in migraine compared with TTH. The pathophysiology of migraine is thought to involve several processes that have been implicated in the generation of reactive oxygen species (ROS) and in the consumption of brain antioxidant reserves, including recurrent trigeminovascular activation with release of calcitonin gene-related peptide (CGRP) and neurogenic inflammation, glutamatergic excitotoxicity, and mitochondrial dysfunction with altered energy metabolism [12, 13, 15]. By contrast, current pathophysiological models of TTH emphasize peripheral myofascial nociception and central pain-modulating dysfunction, and the trigeminovascular and mitochondrial/energy-metabolism mechanisms that have been implicated in migraine appear to be less consistently reported in TTH [4]. In addition, peripheral blood studies have reported elevated oxidative damage markers and reduced antioxidant capacity in migraine patients, whereas comparable alterations appear to have been less consistently reported in TTH, although direct head-to-head comparisons remain limited [13, 14]. Collectively, these considerations raise the possibility that thalamic GSH — the brain’s principal antioxidant buffer — may be reduced in migraine, whereas comparable reductions may not be observed in TTH, which, if confirmed, could represent a neurochemical feature that might help to distinguish the two disorders.

In this study, we sought to assess thalamic GSH levels in migraine patients (with and without aura), TTH patients, and healthy controls using optimized MEGA-PRESS spectroscopy. We hypothesized that migraine patients would show altered GSH levels reflecting oxidative stress involvement, and that this alteration might be specific to migraine rather than common to primary headaches in general. Additionally, we performed sex-stratified analyses given the known sex differences in migraine prevalence and pathophysiology, and evaluated whether thalamic GSH could serve as a candidate biomarker for differentiating migraine from TTH.

## Methods

### Subjects

This cross-sectional study recruited participants from the Department of Neurology, Second Affiliated Hospital of Xiamen Medical College between May 2025 and December 2025. The study protocol was approved by the Institutional Ethics Committee[2024048], and written informed consent was obtained from all participants prior to enrollment. Inclusion criteria for headache patients were: (1) diagnosis of episodic migraine (with or without aura) or episodic TTH according to the International Classification of Headache Disorders, 3rd edition (ICHD-3) criteria; (2) age 18–50 years; (3) headache patients underwent interictal scanning at least 72 h after the last attack, with confirmation of no subsequent attack within 48 h thereafter.

Exclusion criteria included: (1) other neurological or psychiatric disorders; (2) history of head trauma, concurrent secondary headache, medication overuse headache, or other types of primary headache; (3) coexistence of tension-type headache and migraine; (4) long-term use of medications that may affect brain metabolism (including prophylactic migraine medications within 4 weeks); (5) contraindications to MRI. Acute medication use (triptans, NSAIDs, or simple analgesics) was permitted, but all participants were scanned at least 72 h after the last headache attack and any acute medication use. Healthy controls were age- and sex-matched volunteers with no history of recurrent headache (fewer than 1 headache day per month) or neurological disorders.

All participants underwent standardized clinical assessment, including demographic data collection, detailed headache history, and neurological examination. For migraine patients, the following clinical parameters were recorded: disease duration (years), attack frequency (days per month), typical attack duration (hours), pain intensity during attacks (Visual Analog Scale, VAS), Headache Impact Test-6 (HIT-6) score and Acquisition Migraine Disability Assessment (MIDAS) score (for migraine patients only).

### MR spectroscopy

**Fig. 1:**
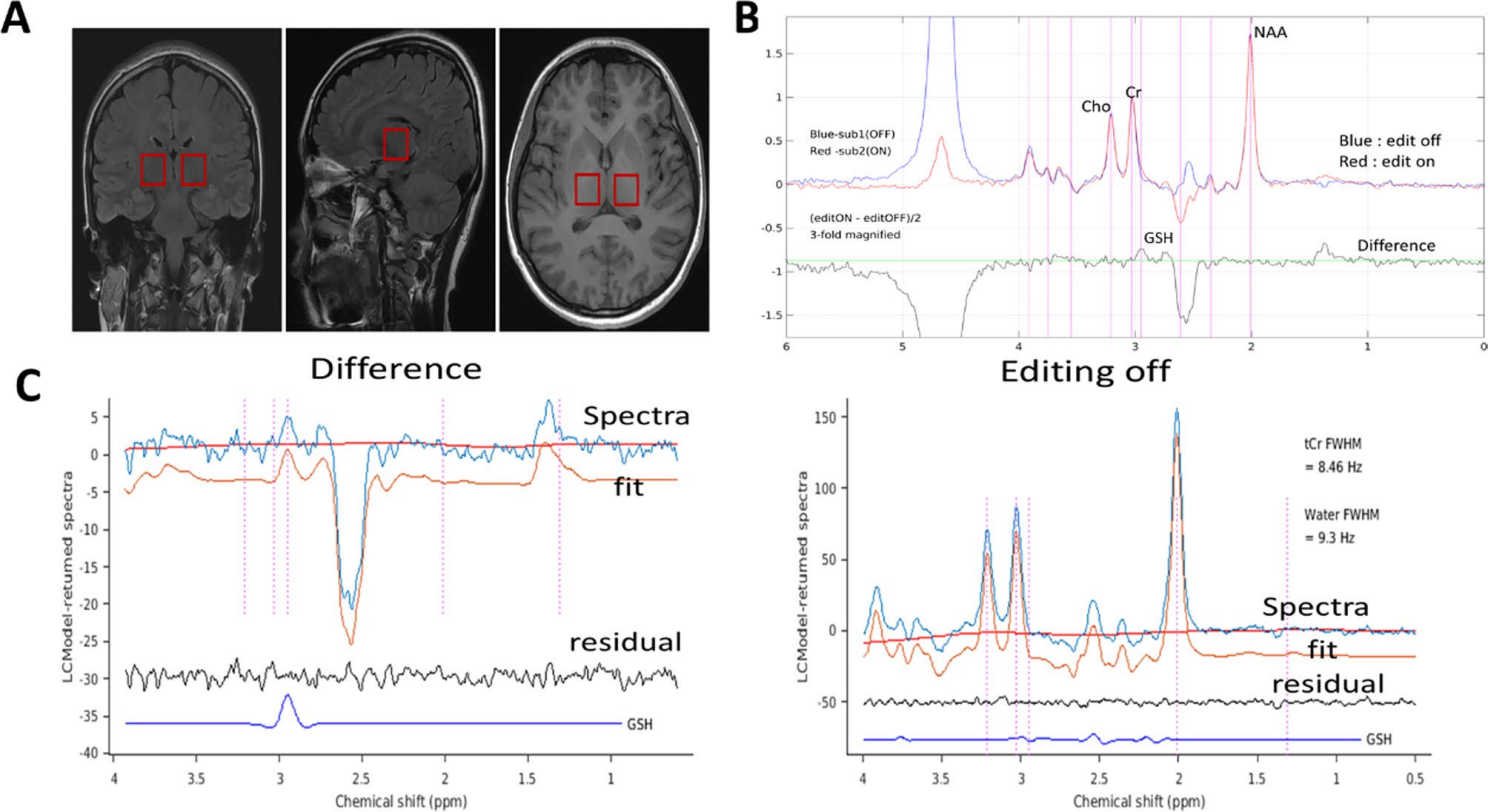
Representative MRS voxel placement, GSH Editing Principle, edited difference spectra and spectral fitting, Editing off spectra and spectral fitting. (**A**) In vivo brain GSH detection using MEGA-PRESS in the left and right thalamus region with size of 20 × 20 × 20 mm. (**B**) GSH Editing Principle, the GSH signal is obtained by (edit ON - edit OFF) / 2. (**C**-**D**) Representative edited Difference spectra and editing OFF spectra from the left thalamus of a migraine patient. The GSH is obtained from the difference spectra, other metabolites are quantified from the Edit-OFF spectra. NAA = N-acetylaspartate, Cr= creatine, Cho= choline, GSH= Glutathione, FWHM= full width at half maximum, ppm= part per million

All MRI and MRS examinations were performed on a clinical 3.0-T MRI scanner (Discovery MR750w, GE Healthcare, Milwaukee, WI, USA) equipped with a 24-channel head coil. The full scan protocol for each session included: (1) a three-plane localizer; (2) tructural MRI data were acquired using a 3D BRAVO (Brain Volume) sequence on a GE MRI scanner, equivalent to a 3D magnetization-prepared rapid gradient echo (MP-RAGE) protocol. Scan parameters were as follows: repetition time (TR) = 6.2 ms, echo time (TE) = 2.3 ms, flip angle = 12°, inversion time (TI) = 450 ms, field of view (FOV) = 240 × 240 mm², acquisition matrix = 256 × 256, slice thickness = 1.50 mm, 96 slices per slab, acquired voxel size = 0.9 × 0.9 × 1.5 mm³, bandwidth = 50.00 kHz, number of excitations (NEX) = 1, frequency encoding direction = anterior/posterior (A/P), and acquisition time approximately 1 min 49 s) for voxel positioning and tissue segmentation; (3) coronal T2-weighted fluid-attenuated inversion recovery (T2 FLAIR) images (TR = 8,000 ms, TE = 120ms, NEX = 1, FOV = 240 × 240 mm², slice thickness = 5 mm, acquisition time = 1 min 44 s); (4) sagittal T2 FLAIR images (TR = 9,000 ms, TE = 145 ms, NEX = 1, FOV = 240 × 240 mm², slice thickness = 5 mm, acquisition time = 1 min 57 s); (5) MEGA-PRESS spectroscopy of the left thalamus; (6) MEGA-PRESS spectroscopy of the right thalamus, during the MEGA-PRESS acquisition, unsuppressed water signals were acquired at the beginning of each block, before the acquisition of water‑suppressed FIDs, for multichannel combination and eddy‑current compensation. The acquisition parameters were: TR = 2000 ms, TE = 79 ms, number of averages = 128 for each of edit-on and edit-off acquisitions, resulting acquisition time of approximately 9 min 20 s per voxel (256 total averages × 2000 ms TR). Water suppression was achieved using the vendor-supplied VAPOR (variable power and optimized relaxation delays) scheme with default parameters. Automated first-order B₀ shimming was performed prior to spectral acquisition. The total scan duration was approximately 27 min per participant. Single-voxel ¹H MRS data were acquired using the MEGA-PRESS sequence for selective detection of glutathione (GSH), following the protocol described previously [24]. To avoid potential interference from the skull, sinus structures, and cerebrospinal fluid during scanning, a volume of interest of 20 × 20 × 20 mm³ was positioned in the left and right thalamus guided by anatomical landmarks based on T₁-weighted images (for voxel placement see Fig. 1A). Two 10-ms frequency-selective Gaussian editing pulses (truncated at 7%, bandwidth 120 Hz) were implemented in the MEGA-PRESS sequence. In edit-on scans, the editing pulses were tuned at 4.56 ppm to selectively refocus the GSH cysteine resonance, enabling detection of the coupled 2.95-ppm resonance in the difference spectrum. In edit-off scans, the editing pulses were turned off.

Stringent quality control criteria were applied: (1) water FWHM (full width at half maximum) < 10 Hz; (2) tCr singlet FWHM < 10 Hz; (3) Cramér-Rao lower bounds (CRLB) for GSH < 10%. Spectra failing any criterion were excluded from analysis. The CRLB returned by LCModel served as the primary measure of fitting reliability for individual metabolites.

### Data processing and quantification

Spectroscopy data were processed following the methods described previously [24]. Individual free-induction decays were frequency-aligned and phase-corrected prior to summation. Edit-on and edit-off spectra were subtracted to generate difference spectra for GSH quantification (for GSH Editing Principle see Fig. 1B). All spectra were apodized with 1-Hz exponential and 1-Hz Gaussian line-broadening functions before Fourier transformation.

### GSH quantification

Spectral fitting of difference spectra was performed using LCModel software [25] with a basis set that included GSH, N-acetylaspartate (NAA) aspartate moiety, creatine CH₂, lactate, threonine, and macromolecular resonances at 1.24 and 1.42 ppm (for representative edited Difference spectra and spectral fitting see Fig. 1C). The basis signals were generated by density-matrix simulations incorporating the actual RF pulse shapes and timing parameters of the MEGA-PRESS sequence. Spectral fitting was performed between 0.2 and 4.0 ppm. We used an internal metabolite referencing method: total creatine (tCr) was used as a reference with its concentration set to 8 mM, and the brain GSH concentration was calculated by normalizing the zero‑TE extrapolated GSH signal to the zero‑TE tCr signal [24]. 

To address potential partial volume effects, tissue segmentation was performed on the 3D T1-weighted BRAVO images using SPM12 (Wellcome Trust Centre for Neuroimaging, London, UK). The MRS voxel was co-registered to the T1-weighted images, and the fractional volumes of gray matter (GM), white matter (WM), and cerebrospinal fluid (CSF) within each voxel were calculated. GSH concentrations were corrected for CSF partial volume using the formula: GSH_corrected = GSH_raw / (1 - f_CSF), where f_CSF represents the CSF fraction within the voxel. This correction accounts for signal dilution from CSF, which contains negligible metabolite concentrations. Tissue fractions were compared across groups using Kruskal-Wallis test to ensure that any observed GSH differences were not driven by differences in voxel composition.

### Quantification of other metabolites

Edit-off spectra were independently analyzed using LCModel to quantify non-edited brain metabolites including total N-acetylaspartate (tNAA), total creatine (tCr), total choline (tCho), glutamate (Glu), glutamine (Gln), and myo-inositol (mI) (for representative editing OFF spectra and spectral fitting see Fig. 1D). The basis set included N-acetylaspartate, N-acetylaspartylglutamate, creatine, phosphocreatine, glycerophosphocholine, phosphocholine, glutamate, glutamine, myo-inositol, γ-aminobutyric acid, glycine, scyllo-inositol, aspartate, taurine, ethanolamine, phosphoethanolamine, glucose, and lactate. The LCModel built-in macromolecular and lipid basis signals were included in the fitting.

### Metabolite concentration estimation

Metabolite concentrations were calculated with reference to total creatine, assuming a tCr concentration of 8 mM [24, 26]. Only metabolite estimates with Cramér-Rao lower bounds (CRLB) ≤ 12% were included in subsequent analyses.

It should be noted that the relatively long TE (79 ms) used in this study was optimized for GSH detection. Metabolites with long T₂ relaxation times (tNAA, tCr, tCho) are reliably quantified from edit-off spectra, whereas metabolites with shorter T₂ values (Glu, Gln, mI) may be subject to T₂-related signal attenuation and are reported as apparent concentrations.

### Statistical analysis

Post-hoc power analysis using G*Power 3.1 confirmed that our sample size (*n* = 76) provided adequate statistical power (> 0.80) to detect the observed effect sizes (partial η² = 0.35) at α = 0.05. Statistical analyses were performed using SPSS version 26.0 (IBM Corp., Armonk, NY, USA). Continuous variables were expressed as mean ± standard deviation (SD). Normality of the data was assessed using the Shapiro–Wilk test. Data normality and homogeneity of variances were verified using Shapiro-Wilk and Levene’s tests.

Group comparisons for thalamic GSH concentrations were performed using one-way ANOVA, with Tukey’s HSD post-hoc tests for pairwise comparisons to control for multiple comparisons. Continuous variables between two independent groups were compared using independent-samples t-tests; tissue fractions were compared across groups using the Kruskal–Wallis test; categorical variables were compared using the Chi-square test; comparisons between the two thalami were performed using a paired t-test. Effect sizes were reported as partial η² for omnibus tests and Cohen’s d for pairwise comparisons. Given sex imbalance across groups, sex-stratified analyses were conducted. Pearson correlations examined relationships between thalamic GSH (left and right thalamus, analysed separately) and clinical variables within the migraine group (five variables: disease duration, attack frequency, VAS, MIDAS, HIT-6) and within the TTH group (four variables: disease duration, attack frequency, VAS, HIT-6; MIDAS is specific to migraine). P-values from correlation analyses were adjusted for multiple comparisons using the Benjamini–Hochberg false discovery rate (FDR) procedure at q = 0.05, applied separately within each group.

ROC curve analysis evaluated the diagnostic performance of thalamic GSH, with AUC, sensitivity, specificity, and optimal cutoff values (Youden’s index) calculated.

## Results

### Participant characteristics

A total of 82 participants were initially recruited. Six participants were excluded due to spectral quality issues, leaving 76 participants for analysis: 20 healthy controls (HC), 19 TTH patients, and 37 migraine patients (27 without aura, 10 with aura). 55 participants had bilateral thalamic data, while 21 participants had only left thalamic data due to time constraints or positioning issues with the right thalamic voxel. The demographic and clinical characteristics are summarized in Table 1. The three groups were matched for age (*p* = 0.966). The migraine group had a higher proportion of females (75.7%) compared to HC (50.0%) and TTH (63.2%), consistent with known epidemiology, though this difference did not reach statistical significance (*p* = 0.144). Among headache patients, migraine patients had significantly higher pain intensity (*p* < 0.001) and HIT-6 scores (*p* = 0.008) compared to TTH patients, reflecting greater headache-related disability.

Spectral quality metrics and voxel tissue composition across groups are summarized in Table 2. The mean water linewidth was 9.13 ± 0.88 Hz and the mean tCr linewidth was 8.49 ± 0.82 Hz across all included spectra, confirming that all data met the quality thresholds. The CRLB returned by LCModel served as the primary measure of fitting reliability for individual metabolites. Tissue segmentation analysis revealed that voxel composition was comparable across groups for the left thalamus: GM fraction, WM fraction, and CSF fraction and right thalamus (all *p* > 0.10). The absence of significant differences in tissue composition across groups indicates that the observed GSH differences are not attributable to partial volume effects.

**Table 1:** Demographic and clinical characteristics of study participants

Characteristic	HC (*n* = 20)	TTH (*n* = 19)	Migraine (*n* = 37)	*p*-value
Age, years	30.6 ± 13.4	30.5 ± 11.8	29.9 ± 10.9	0.966^a^
Sex, Female/Male	10/10	12/7	28/9	0.144^b^
Female proportion (%)	50.0%	63.2%	75.7%	-
Bilateral data available, n (%)	16 (80%)	13 (68%)	26 (70%)	0.672^b^
Disease duration, years	-	5.2 ± 3.8	7.1 ± 5.4	0.156^c^
Attack frequency, days/month	-	4.8 ± 2.9	5.6 ± 3.7	0.421^c^
Pain intensity (VAS, 0–10)	-	4.2 ± 1.6	6.8 ± 1.9	< 0.001^c***^
HIT-6 score	-	52.1 ± 7.3	59.8 ± 8.2	0.008^c**^
With aura, n (%)	-	-	10 (27%)	-

Data expressed as mean ± SD or n (%). HC: healthy control; TTH: tension-type headache; VAS: visual analog scale; HIT-6: Headache Impact Test-6

^a^One-way ANOVA; ^b^Chi-square test; ^c^Independent samples t-test

**p < 0.01, ***p < 0.001

**Table 2:** Spectral quality across groups and voxel tissue composition

Parameter	HC(*n* = 20)	TTH(*n* = 19)	Migraine(*n* = 37)	*p*-value
tCr FWHM (Hz)	8.78 ± 0.91	8.46 ± 0.76	8.55 ± 0.79	0.12^a^
Water FWHM (Hz)	9.33 ± 0.88	9.17 ± 0.94	9.00 ± 0.83	0.18^a^
***Left thalamus***				
fGM	0.15 ± 0.03	0.14 ± 0.05	0.14 ± 0.04	0.59^b^
fWM	0.85 ± 0.03	0.86 ± 0.05	0.86 ± 0.04	0.59^b^
fCSF	0.00 ± 0.00	0.00 ± 0.00	0.00 ± 0.00	0.12^b^
***Right thalamus***				
fGM	0.16 ± 0.12	0.12 ± 0.04	0.12 ± 0.04	0.55^b^
fWM	0.82 ± 0.18	0.88 ± 0.04	0.88 ± 0.04	0.53^b^
fCSF	0.02 ± 0.06	0.00 ± 0.00	0.00 ± 0.00	0.77^b^

Abbreviations: HC, healthy controls; TTH, tension-type headache; fGM, gray matter fraction; fWM, white matter fraction; fCSF, cerebrospinal fluid fraction

Values are presented as mean ± SD

^a^ One-way ANOVA; ^b^ Kruskal-Wallis test

No significant differences were observed among groups (all *p* > 0.05)

### Thalamic GSH levels

**Fig. 2:**
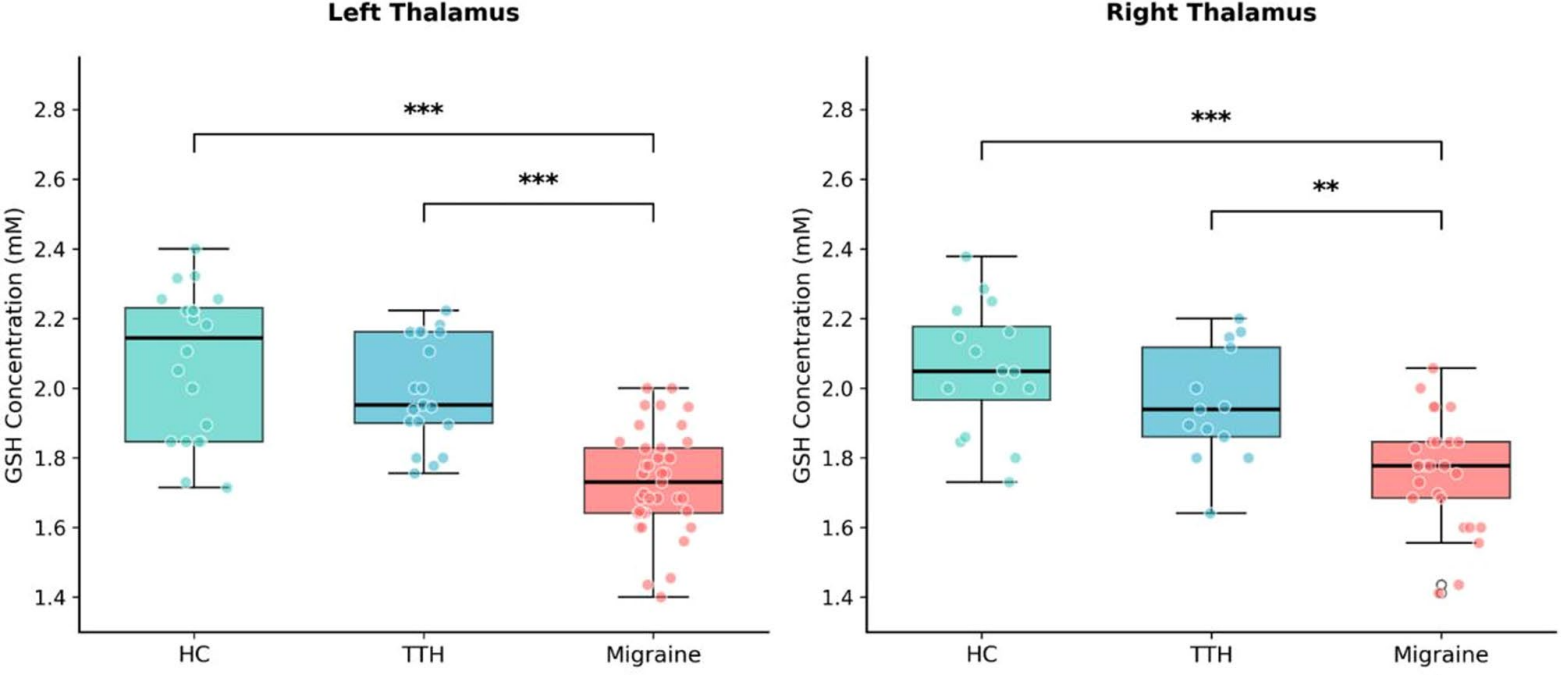
Thalamic GSH concentrations across groups. Box plots showing GSH levels in the left and right thalamus for healthy controls (HC), tension-type headache (TTH), and migraine groups. ***p < 0.001, **p < 0.01

Thalamic GSH concentrations differed significantly among groups in both hemispheres (Fig. 2). Shapiro-Wilk tests confirmed normal distribution for all groups (all *p* > 0.05), and Levene’s tests confirmed homogeneity of variances, supporting the use of parametric statistics. In the left thalamus, one-way ANOVA revealed a highly significant group effect (F(2,73) = 30.78, *p* < 0.001, η² = 0.457). GSH levels were highest in HC (2.07 ± 0.22 mM), intermediate in TTH (1.99 ± 0.15 mM), and lowest in migraine (1.73 ± 0.15 mM). Similar findings were observed in the right thalamus (F(2,52) = 16.26, *p* < 0.001, η² = 0.385). Tukey HSD post-hoc comparisons revealed that migraine patients had significantly lower GSH than both HC (left: t = 7.01, *p* < 0.001, Cohen’s d = 1.95; right: t = 5.48, *p* < 0.001, d = 1.74) and TTH patients (left: t = 6.05, *p* < 0.001, d = 1.71; right: t = 3.50, *p* = 0.004, d = 1.19). Critically, no significant difference was observed between HC and TTH groups in either hemisphere (left: *p* = 0.288; right: *p* = 0.247).

### Specificity of GSH alterations

To confirm the specificity of GSH changes, we examined other thalamic metabolites from edit-off spectra including glutamate (Glu), myo-inositol (mI), total choline (tCho), N-acetylaspartate (NAA), NAAG, and total NAA (tNAA). None of these metabolites showed significant group differences in either hemisphere (all F < 1.0, all *p* > 0.05).

### Bilateral symmetry

Paired analysis of participants with bilateral data (*n* = 55) revealed no significant left-right asymmetry in GSH levels within any group (Table 3). The mean differences between left and right thalamic GSH were small and non-significant in all groups (HC: *p* = 0.570; TTH: *p* = 0.301; Migraine: *p* = 0.330), indicating that GSH reduction in migraine affects both hemispheres symmetrically.

**Table 3:** Bilateral comparison of thalamic GSH levels

Group	*n* (pairs)	Left Thalamus (mM)	Right Thalamus (mM)	Difference (L-*R*)	*p*-value
HC	16	2.03 ± 0.21	2.06 ± 0.18	-0.035 ± 0.266	0.570
TTH	13	2.02 ± 0.16	1.95 ± 0.17	0.065 ± 0.240	0.301
Migraine	26	1.72 ± 0.14	1.76 ± 0.16	-0.038 ± 0.159	0.330

Data expressed as mean ± SD. Paired samples t-test. No significant left-right asymmetry was observed in any group

### Sex-stratified analysis

**Fig. 3:**
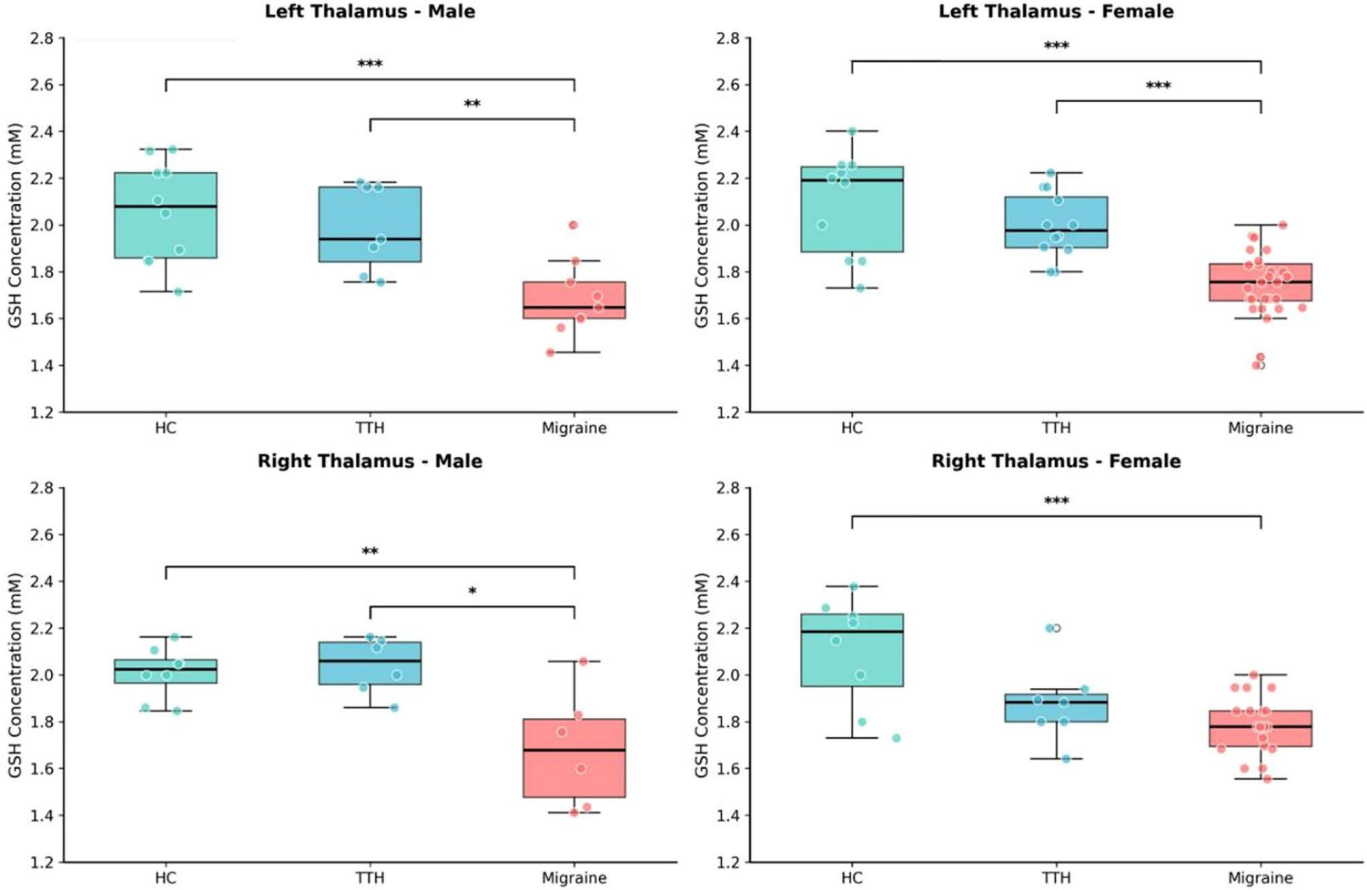
Sex-stratified analysis of thalamic GSH concentrations. Box plots showing GSH levels stratified by sex (male and female) and brain region (left and right thalamus). ***p < 0.001, **p < 0.01, *p < 0.05

Given the well-known sex differences in migraine prevalence and the sex imbalance in our cohort, we performed sex-stratified analyses to confirm that GSH differences were not confounded by sex (Fig. 3). Within each group, no significant sex differences in GSH levels were observed (all *p* > 0.05). In males, significant group differences persisted in both hemispheres (left: F = 9.53, *p* = 0.001, η² = 0.453; right: F = 8.89, *p* = 0.002, η² = 0.511). Similarly, in females, group differences remained highly significant (left: F = 21.04, *p* < 0.001, η² = 0.472; right: F = 10.83, *p* < 0.001, η² = 0.404). These results suggest that thalamic GSH reduction in migraine is consistent across both sexes.

### Diagnostic performance

**Fig. 4:**
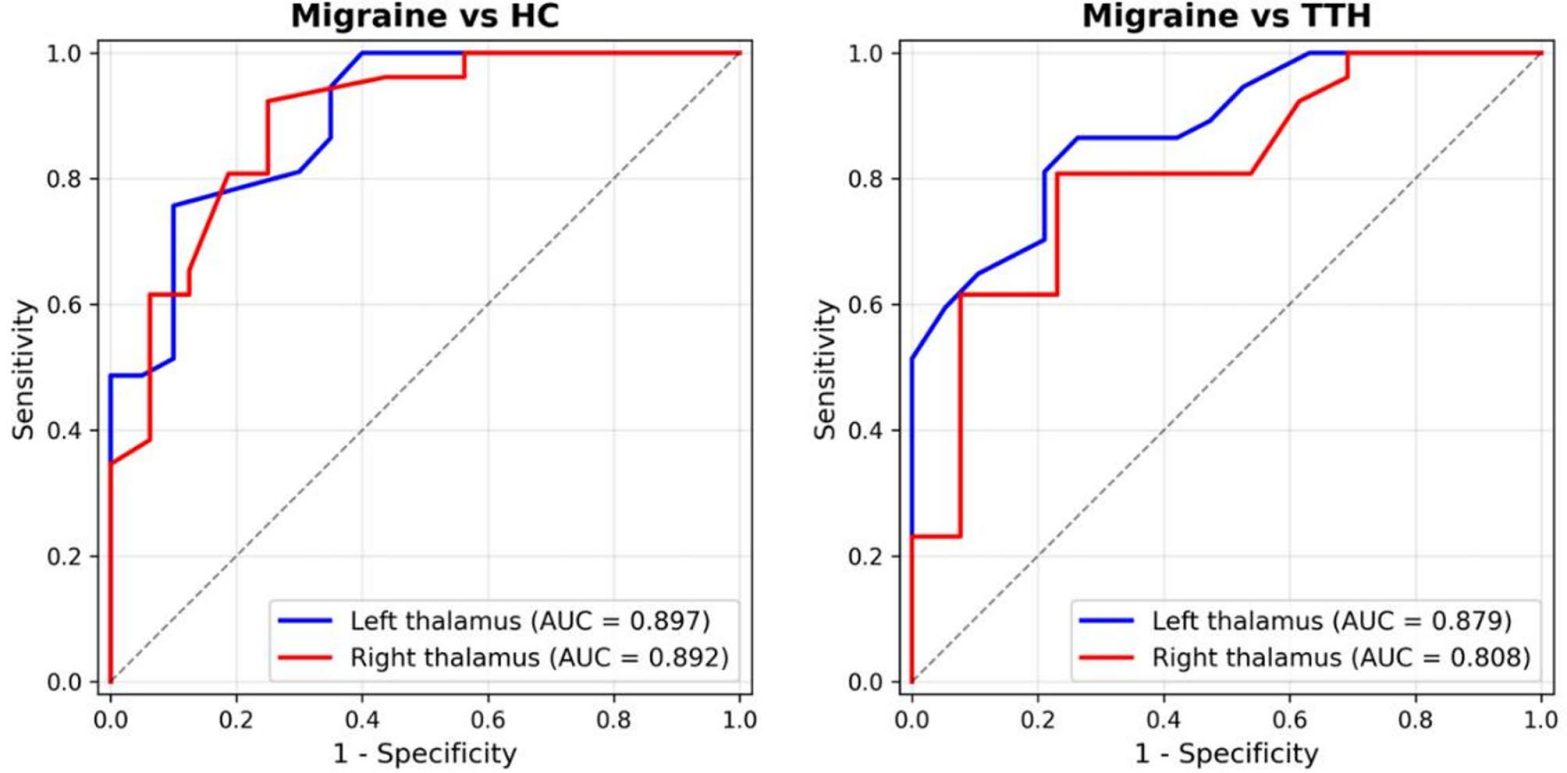
ROC curves for differentiating migraine from HC (**A**) and TTH (**B**) using thalamic GSH. Each panel shows curves for both left thalamus (blue) and right thalamus (red). AUC= area under the curve

ROC analysis showed that thalamic GSH had good diagnostic accuracy for distinguishing migraine from both HC and TTH (Fig. 4). For differentiating migraine from HC, the left thalamus showed excellent performance with AUC of 0.897 (95% CI: 0.801–0.965); the optimal cutoff of GSH ≤ 1.829 mM yielded sensitivity of 75.7% and specificity of 90.0%. The right thalamus showed similar performance (AUC = 0.892, 95% CI: 0.774–0.978; optimal cutoff ≤ 1.946 mM, sensitivity 92.3%, specificity 75.0%). For the clinically more relevant comparison of migraine versus TTH, the left thalamus achieved AUC of 0.879 (95% CI: 0.780–0.958); the optimal cutoff of GSH ≤ 1.895 mM yielded sensitivity of 86.5%, specificity of 73.7%, and overall accuracy of 80.1%. The right thalamus showed slightly lower but still acceptable performance (AUC = 0.808, 95% CI: 0.647–0.933; optimal cutoff ≤ 1.846 mM, sensitivity 80.8%, specificity 76.9%).

### Correlations with clinical variables

Pearson correlation analyses examined relationships between thalamic GSH concentrations and clinical severity indices. In the migraine group, no significant correlations were observed between left thalamic GSH and disease duration (*r* = − 0.12, *p* = 0.48), attack frequency (*r* = − 0.08, *p* = 0.64), pain intensity (*r* = − 0.15, *p* = 0.37), MIDAS score (*r* = − 0.18, *p* = 0.29), or HIT-6 score (*r* = − 0.14, *p* = 0.41) after FDR correction; right thalamic GSH showed a comparable null pattern (all |r| < 0.20, all FDR-corrected *p* > 0.25). In the TTH group, the four applicable clinical variables (disease duration, attack frequency, VAS, HIT-6; MIDAS is specific to migraine) likewise showed no significant correlation with thalamic GSH after FDR correction (all |r| < 0.25, all FDR-corrected *p* > 0.30).

## Discussion

The present study examined thalamic GSH levels in migraine, tension-type headache (TTH), and healthy controls using MEGA-PRESS spectroscopy. Thalamic GSH was lower in migraine patients than in both healthy controls and TTH patients, while GSH levels in TTH did not differ from those of healthy controls. In addition, sex-stratified analyses showed that the reduction in migraine was observed in both sexes, and left thalamic GSH showed acceptable diagnostic performance for separating migraine from TTH. These observations are consistent with, though do not prove, a role for oxidative stress in migraine pathophysiology, and are discussed below in the context of existing literature and the limitations of the present sample size.

### GSH reduction as a migraine-specific finding

The most notable finding is the apparent differentiation of migraine from TTH based on GSH levels. Migraine patients showed approximately 16% reduction in thalamic GSH compared to healthy controls, with large effect sizes (Cohen’s d > 1.7). In contrast, TTH patients showed GSH levels that were not significantly different from controls (difference < 4%). This pattern was consistent across both hemispheres and remained robust after correction for multiple comparisons. However, this interpretation should be viewed with caution given the modest TTH sample size (*n* = 19), which may have limited statistical power to detect smaller GSH alterations in this group. Additionally, the TTH group may encompass heterogeneous subtypes (e.g., infrequent vs. frequent episodic TTH) that could have differential effects on oxidative stress markers; our study was not powered to examine such subgroup differences.

Our results extend previous MRS studies reporting metabolite alterations in migraine. Bathel et al. observed increased glutamate/glutamine (GLX) levels in the thalamus and occipital cortex of migraine patients without aura, supporting the notion of an extended network displaying cortical hyperexcitability [10]. Mohamed et al. also reported interictal alterations of thalamic metabolic concentration ratios in migraine without aura [27]. More recently, Zhang et al. reported changes in GABA and Glx levels in the right thalamus of chronic migraine patients [11]. The present study adds oxidative stress, as indexed by GSH depletion, as another dimension of thalamic involvement in migraine. Notably, we found that other metabolites (Glu, mI, tCho, NAA, tNAA) did not differ among groups, paralleling Bathel’s finding that GABA was unchanged in episodic migraine, and highlighting the selectivity of neurochemical alterations in migraine.

The finding that TTH patients show normal GSH levels is particularly important for understanding pathophysiological distinctions between these conditions. While some researchers have proposed that migraine and TTH exist on a continuum [28], our data support the view that these conditions have distinct underlying mechanisms, at least with respect to oxidative stress involvement. This is consistent with recent findings by Vuralli et al. showing that somatosensory temporal discrimination is disrupted in migraine but remains intact in TTH, further supporting the notion of distinct pathophysiological mechanisms [29]. This distinction has practical implications for differential diagnosis and potentially for treatment selection.

### Comparison with prior GSH studies in migraine

To our knowledge, this is the first study to specifically measure thalamic GSH levels in migraine patients using MRS. Prior MRS studies of GSH in migraine have focused primarily on the occipital cortex. Zhang et al. reported that occipital GSH levels were reduced during migraine attacks compared to the interictal period in migraine without aura patients, indicating dynamic changes in oxidative stress across the migraine cycle [23]. Notably, their study found no significant difference in interictal occipital GSH between migraine patients and healthy controls, which the authors took to suggest that GSH changes in the occipital cortex may be more evident during attacks than in the interictal phase. By contrast, our observation of reduced thalamic GSH during the interictal period, if confirmed in larger cohorts, would point to a more persistent alteration in this region.

The discrepancy between our robust finding of reduced thalamic GSH and the less consistent findings in the occipital cortex raises important questions about regional specificity. One possibility is that GSH reduction is particularly pronounced in the thalamus due to its central role in nociceptive processing and pain transmission, as discussed below. The thalamus receives direct input from the trigeminovascular system during every migraine attack and may therefore be subject to greater cumulative oxidative stress than cortical regions less directly involved in pain processing. Alternatively, methodological differences including field strength, voxel placement, and acquisition parameters may contribute to the variability across studies.

Future multi-region MRS studies examining GSH simultaneously in the thalamus, occipital cortex, and other brain regions within the same patients would help clarify whether GSH reduction is specific to the thalamus or represents a more widespread phenomenon. Such studies would have important implications for understanding whether oxidative stress in migraine preferentially affects regions involved in trigeminal nociception and central pain processing, or reflects a more generalized metabolic vulnerability. As a limitation, the current study examined only the thalamus, precluding direct conclusions about regional specificity.

### Thalamic involvement in migraine pathophysiology

The thalamus occupies a pivotal position in migraine pathophysiology, serving as the principal relay station for ascending nociceptive signals from the trigeminovascular system to the cortex. Our finding of reduced thalamic GSH in migraine patients, obtained during the interictal phase, suggests that a neurochemical alteration in this region may be detectable even in the absence of an ongoing attack. A growing body of neuroimaging work in migraine without aura nonetheless suggests that the thalamus is not silent between attacks. Voxel-based morphometry studies have reported subtle grey-matter alterations in thalamic subnuclei in interictal episodic migraine [8]. Diffusion tensor imaging studies in migraine without aura have described thalamic microstructural changes between attacks — in particular increased fractional anisotropy that varies with time since the last attack and appears to normalise during the attack itself [30] — and correlated structural—functional alterations of the thalamo-cortical network [31]. Resting-state fMRI studies of migraine without aura during the interictal phase have, in turn, reported altered intrinsic connectivity between the thalamus and the visual cortex [32], altered connectivity of specific thalamic subregions, including the anterior dorsal and ventral posterior nuclei, with the precuneus and pain-processing cortices [33], and abnormal dynamic thalamocortical network states [34]. These structural and functional observations have been integrated under the broader concept of thalamocortical dysrhythmia, in which persistent alterations in thalamic output and thalamocortical rhythms are thought to contribute to interictal features of migraine such as deficient habituation and multisensory hypersensitivity [35]. Within this framework, our observation of reduced thalamic GSH during the interictal phase may be regarded as a candidate neurochemical correlate of these chronic, between-attack thalamic changes, possibly reflecting cumulative oxidative burden in a region chronically exposed to trigeminovascular input over years of recurrent attacks.

Several non-mutually-exclusive mechanisms may plausibly contribute to the reduced interictal thalamic GSH observed in our study. First, migraine has been increasingly conceptualised as a disorder of brain energy metabolism, in which mitochondrial dysfunction and impaired oxidative phosphorylation are reported between attacks rather than only during them [12, 15, 36, 37]. Chronic mitochondrial inefficiency is an established source of continuous reactive oxygen species generation and can further compromise GSH synthesis and regeneration through effects on ATP production and the γ-glutamyl cycle [38]. A mitochondrial and metabolic substrate therefore represents a plausible upstream explanation for reduced interictal thalamic GSH. Second, repeated migraine attacks are accompanied by ictal oxidative stress and transient antioxidant consumption [39]. If GSH is not fully replenished between attacks, the levels measured during the interictal phase may, in part, reflect the accumulated biochemical footprint of prior attacks rather than any process that is active at the moment of scanning.

Third, as outlined above, interictal neuroimaging studies have described persistent structural and functional alterations of the thalamus and thalamo-cortical networks in migraine without aura [8, 34, 31, 35]. A chronically altered thalamic microenvironment — whether reflecting subtle neuronal, glial, or microvascular changes — could plausibly be associated with a shifted oxidative / antioxidant balance that is detectable as lower GSH.

These mechanisms are speculative and are offered to illustrate the range of plausible explanations for the reduced interictal thalamic GSH observed in our study. In this context, Zielman et al. reported elevated cortical glutamate levels in migraine patients using 7T MRS, suggesting that excitotoxicity-related oxidative stress may further contribute to antioxidant depletion [40]. The absence of correlation between GSH levels and disease duration or attack frequency in our data is compatible with the possibility that GSH depletion represents an early feature of migraine rather than a progressive consequence of accumulated attacks; however, given the cross-sectional design and modest sample size, this interpretation remains tentative. Disentangling these mechanisms will require longitudinal MRS combined with structural, functional, and metabolic imaging performed in the same individuals across different phases of the migraine cycle. Reduced thalamic GSH and increased oxidative stress may affect the local neurochemical environment of the thalamus through several interconnected mechanisms. First, reduced antioxidant capacity increases neuronal vulnerability to glutamate-mediated excitotoxicity; oxidative stress can potentiate NMDA receptor function while impairing astrocytic glutamate reuptake [41, 42]. Second, reactive oxygen species (ROS) can directly activate transient receptor potential (TRP) channels, including TRPA1 and TRPV1, which are expressed on thalamic neurons [43]. Third, oxidative stress promotes neuroinflammation through activation of redox-sensitive transcription factors such as NF-κB, leading to increased pro-inflammatory cytokine production [39]. Together, these mechanisms may alter the neurochemical milieu of the thalamus in migraine patients.

Altered thalamocortical connectivity has also been reported in functional neuroimaging studies of migraine patients. Enhanced connectivity between the thalamus and sensory and limbic cortices has been observed [34], and may underlie clinical features of migraine such as photophobia and phonophobia. Our observation of reduced thalamic GSH is consistent with the possibility that altered redox balance in this region may contribute to aberrant thalamocortical signaling.

The bilateral and symmetric nature of GSH reduction observed in our study is consistent with the bilateral involvement of the thalamus in migraine pathophysiology and suggests a systemic metabolic vulnerability rather than a lateralized structural abnormality.

### Sex differences and GSH

Our sex-stratified analyses revealed that thalamic GSH reduction in migraine was significant and comparable in both males (η² = 0.453–0.511) and females (η² = 0.404–0.472). This finding is noteworthy given the well-known female predominance in migraine (3:1 female-to-male ratio) and the suggestion that sex hormones may modulate oxidative stress pathways [44, 45]. The consistency of GSH reduction across sexes suggests that oxidative stress involvement in migraine is not primarily driven by sex-specific factors, though our study was not powered to detect subtle sex differences in effect magnitude.

### Clinical implications

The diagnostic performance of thalamic GSH (AUC = 0.879) in this exploratory analysis suggests it warrants further investigation as a potential candidate diagnostic marker. While migraine diagnosis currently relies on clinical criteria, validation of these findings in independent cohorts is essential before any clinical application can be considered [46]. The optimal cutoff of GSH ≤ 1.895 mM achieved 86.5% sensitivity and 73.7% specificity, comparing favorably with other proposed migraine markers and with recently reported AUC values for thalamic GABA in differentiating chronic migraine from controls (AUC = 0.83) [11]. 

From a therapeutic perspective, GSH reduction suggests that antioxidant strategies may be beneficial in migraine. N-acetylcysteine (NAC), a GSH precursor that crosses the blood-brain barrier, has shown promise in preliminary studies and warrants further investigation as a preventive treatment [47, 48]. Other nutraceuticals with antioxidant properties, including riboflavin, coenzyme Q10, and alpha-lipoic acid, have shown efficacy in migraine prevention and may act in part by supporting antioxidant defenses [15, 49]. 

Our correlation analyses did not reveal a significant association between thalamic GSH and any of the tested clinical variables after FDR correction (all |r| ≤ 0.25 across groups and hemispheres). These null findings are broadly consistent with those of Zhang et al. [23], who likewise reported no significant correlation between occipital GSH and disease duration, VAS, MIDAS, or HIT-6 in migraine without aura. Taken together with our results, this pattern suggests that — at least with the current sample sizes and using the available clinical severity indices — a close coupling between brain GSH levels and these symptom-based measures has not been established. Any diagnostic or therapeutic inference drawn from the group-level GSH reduction therefore remains hypothesis-generating, and would need to be confirmed in larger, independent cohorts using longitudinal designs and ideally more objective indices of disease activity, such as prospective headache diaries or attack-phase measurements.

### Limitations

Several limitations should be noted. First, the cross-sectional design cannot establish causality—whether GSH reduction predisposes to migraine or results from the disorder remains unclear. Longitudinal studies measuring GSH during and between attacks are needed [50]. Second, the TTH sample (*n* = 19) may have been underpowered to detect subtle GSH alterations, and the conclusion that GSH reduction is specific to migraine should be interpreted cautiously pending replication with larger TTH cohorts that account for potential heterogeneity (e.g., episodic frequency, coexisting conditions). Third, MRS measures total tissue GSH and cannot distinguish between neuronal and glial compartments. Furthermore, the 20 × 20 × 20 mm³ voxel encompasses multiple thalamic nuclei involved in different aspects of sensory processing; the current spatial resolution does not allow differentiation of GSH levels among specific nuclei such as the VPM, VPL, or pulvinar, and future studies with higher spatial resolution or multi-voxel approaches may help delineate nucleus-specific GSH alterations. Additionally, MEGA-PRESS GSH quantification presents inherent methodological challenges: the relatively long TE (79 ms) required for J-difference editing results in T₂-weighted signal attenuation, and the low concentration of GSH (~ 1–2 mM) combined with spectral overlap necessitates careful interpretation of absolute values; however, these factors would affect all groups equally and do not invalidate between-group comparisons. Fourth, the interictal measurement timing means we cannot comment on GSH dynamics during attacks. Fifth, although preventive medications were excluded, acute medication use (triptans, NSAIDs, or simple analgesics) was permitted with a minimum 72-hour washout period before scanning; while unlikely to significantly affect interictal GSH levels given the short half-lives of these medications, we cannot completely rule out residual pharmacological effects. Sixth, the ROC analysis and diagnostic cutoff values derived from our data were not validated in an independent external cohort; prospective validation studies are essential before thalamic GSH can be considered a clinically useful biomarker for migraine diagnosis. Finally, while our groups were matched for age, the sex imbalance (higher female proportion in migraine) reflects epidemiology but could introduce confounding; our sex-stratified analyses help address this concern; we did not collect data on hormonal status, including menstrual cycle phase or oral contraceptive use, which may influence GSH levels given the known interactions between sex hormones and oxidative stress pathways; future studies should account for these factors. Given these limitations, replication of our findings in larger, multicenter cohorts with diverse patient populations is strongly encouraged to confirm the generalizability and clinical utility of thalamic GSH as a migraine biomarker.

## Conclusions

We report reduced thalamic GSH levels in migraine patients, with GSH levels in TTH patients that were not significantly different from healthy controls. This exploratory, cross-sectional finding suggests a distinct role of oxidative stress in migraine pathophysiology. ROC analysis indicated that thalamic GSH may have potential to differentiate migraine from TTH, but these results require validation in independent cohorts. Sex-stratified analyses showed that GSH reduction is consistent in both males and females. Further research is needed to investigate GSH dynamics longitudinally, validate these observations in independent cohorts, explore the therapeutic potential of antioxidant interventions, and confirm these findings in multicenter studies.

## Data Availability

Data are available from the corresponding author upon reasonable request.

## Abbreviations

GSH: Glutathione
TTH: Tension-type headache
HC: Healthy control
MRS: Magnetic resonance spectroscopy
MEGA-PRESS: Mescher-Garwood Point Resolved Spectroscopy
ROC: Receiver operating characteristic
AUC: Area under the curve
MIDAS: Migraine Disability Assessment
HIT-6: Headache Impact Test-6
CRLB: Cramér-Rao lower bounds
FWHM: Full width at half maximum
Glu: Glutamate
mI: Myo-inositol
tCho: Total choline
NAA: N-acetylaspartate
tNAA: Total NAA
ROS: Reactive oxygen species
NAC: N-acetylcysteine
